# Supplementary material for: Increased striatal activity in adolescence benefits learning
Source: Nat Commun. 2017 Dec 19;8:1983. doi: 10.1038/s41467-017-02174-z (PMC5736614; doi:10.1038/s41467-017-02174-z)
Supplement: Supplementary file 1 — Supplementary Information [file 41467_2017_2174_MOESM1_ESM.pdf]

Supplementary Informtation

Supplementary Figures

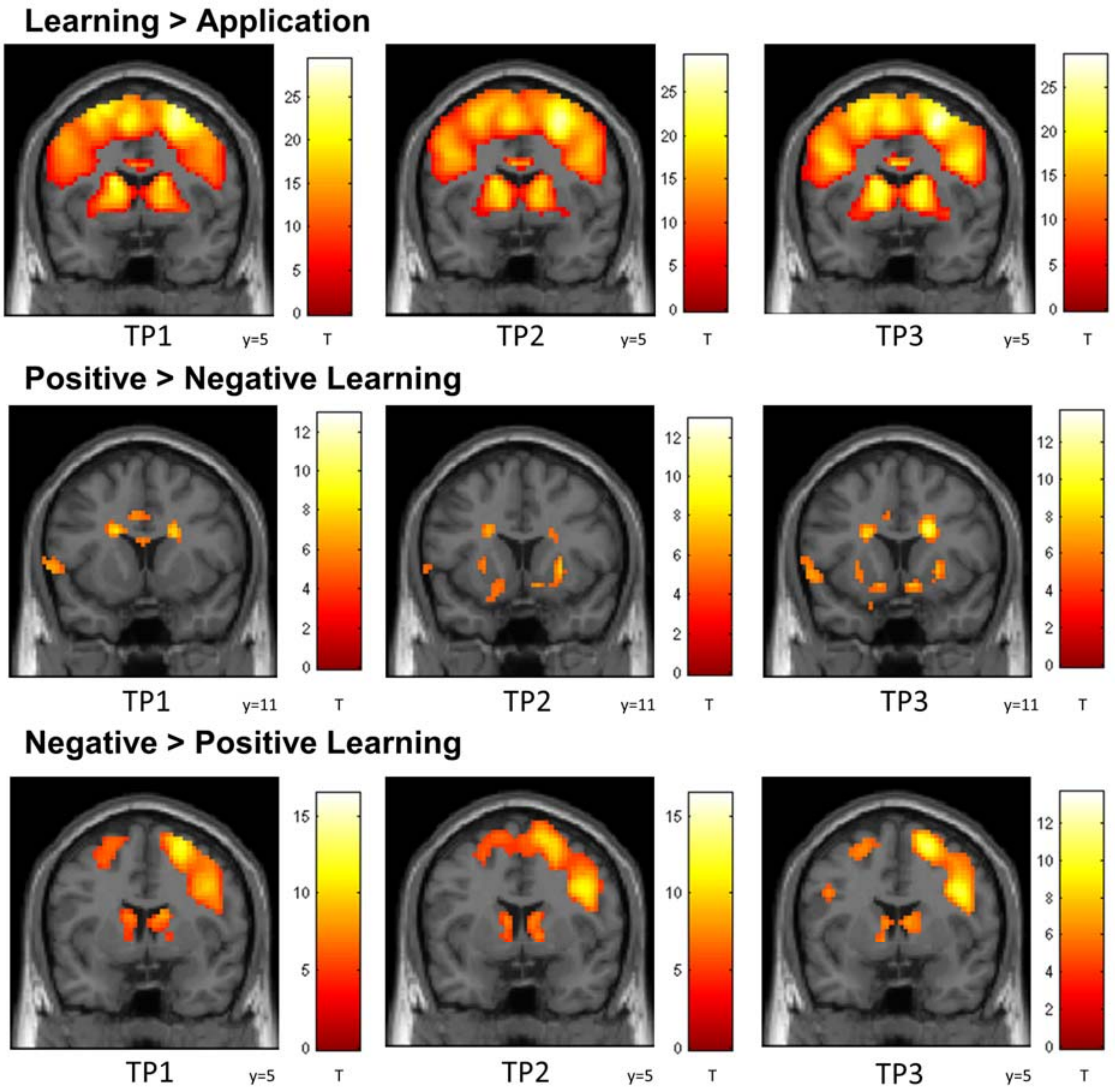

Supplementary Figure 1: Whole-brain results for the contrast learning > application, positive > negative learning and negative > positive learning a TP1, TP2 and TP3. Results are FWE-corrected, *p*<.05. Color bars indicate T-values.

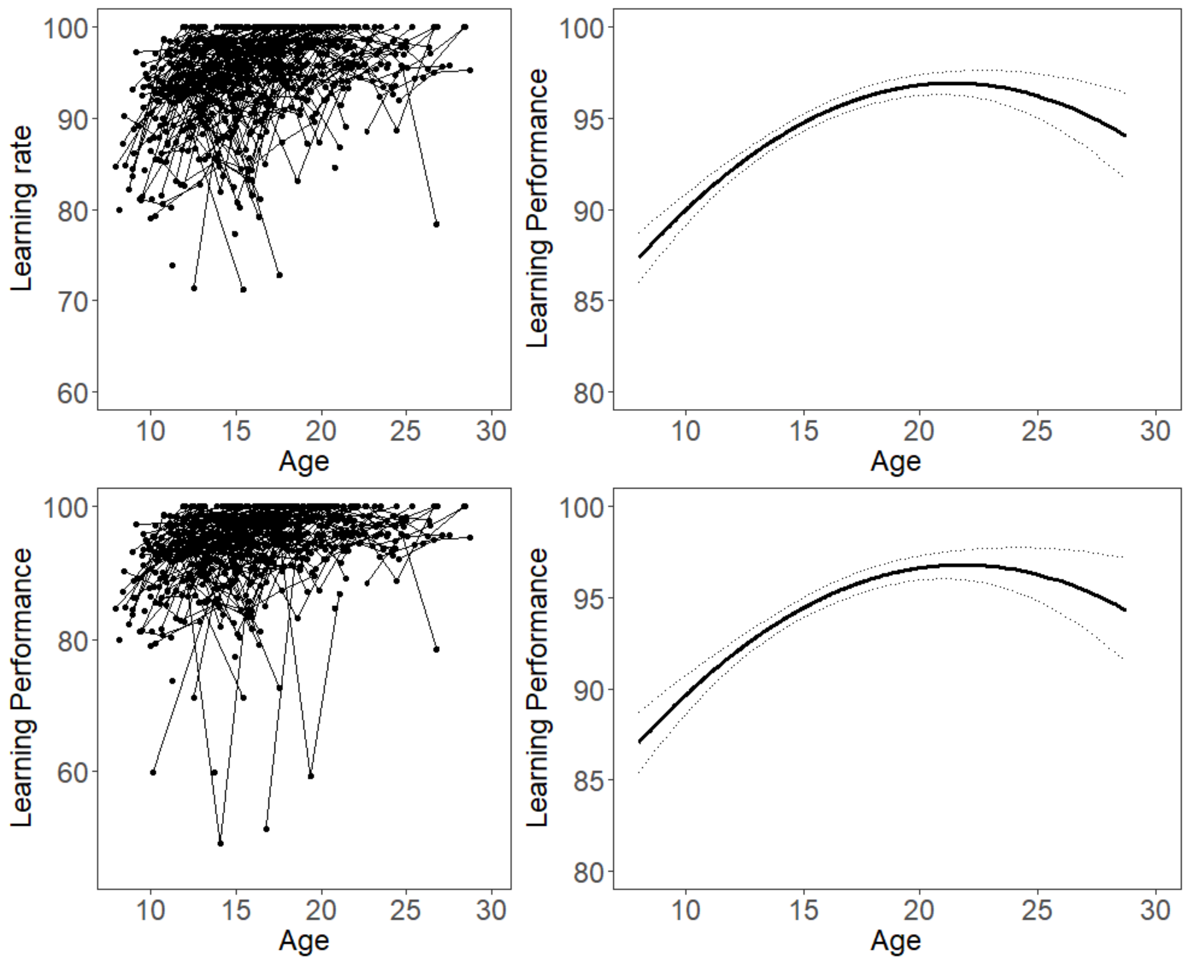

Supplementary Figure 2: Raw and predicted data for learning performance (% of feedback during the learning phase successfully applied in the next trial). The top figures show the data when excluding extreme behavioral outliers (N=5), and the bottom figures show results including behavioral outliers. For both analyses, learning performance was best described by a quadratic trajectory with age.

Supplementary Tables

Supplementary Table 1: MNI-coordinates for local maxima for the sensitivity to informative value (learning > application) and sensitivity to valence (positive > negative learning). The Automated Anatomical Labeling atlas (AAL) was used for labeling of coordinates.

**Learning > Application**	**# voxels**	**T**	**x y z**	**Region**
**TP1**				
	17516	29.31	0 20 46	L Posterior-Medial Frontal Gyrus
		26.87	45 29 34	R Middle Frontal Gyrus
		26.14	24 8 61	R Superior Frontal Gyrus
		26.11	33 20 1	no label
		25.98	27 2 58	R Superior Frontal Gyrus
		25.77	45 -46 52	R Inferior Parietal Lobule
		25.70	48 -43 49	R Inferior Parietal Lobule
		25.55	42 -43 49	R Inferior Parietal Lobule
		25.38	39 -52 52	R Inferior Parietal Lobule
		24.29	-48 -43 49	L Inferior Parietal Lobule
	123	11.33	-66 -31 -5	L Middle Temporal Gyrus
		9.05	-57 -37 -8	L Middle Temporal Gyrus
	120	9.90	48 -28 -5	no label
		9.05	63 -43 -2	R Middle Temporal Gyrus
		8.56	57 -28 -2	R Middle Temporal Gyrus
**TP2**				
	25648	29.19	45 -43 49	R Inferior Parietal Lobule
		28.25	3 17 49	L Posterior-Medial Frontal Gyrus
		26.68	33 20 1	no label
		26.29	27 2 58	R Superior Frontal Gyrus
		25.87	33 -58 49	R Angular Gyrus
		25.55	-30 23 1	L Insula Lobe
		24.68	-48 -43 49	L Inferior Parietal Lobule
		24.03	30 -67 49	R Angular Gyrus
		22.33	-30 -61 49	L Superior Parietal Lobule
		22.28	-33 -58 52	L Inferior Parietal Lobule
	28	7.15	3 -31 28	no label
**TP3**				
	22617	28.54	3 17 49	L Posterior-Medial Frontal Gyrus
		28.07	33 23 1	R Insula Lobe
		27.92	27 2 58	R Superior Frontal Gyrus
		27.20	36 -52 52	R Inferior Parietal Lobule

		27.11	-30	23	1	L Insula Lobe
		26.91	42	-49	49	R Inferior Parietal Lobule
		26.75	-36	-49	49	L Inferior Parietal Lobule
		26.64	33	-55	49	R Inferior Parietal Lobule
		26.58	45	-40	49	R Inferior Parietal Lobule
		25.41	42	29	34	R Middle Frontal Gyrus
**Positive > negative learning**						
**TP1**						
	10376	12.65	-6	-52	13	L Precuneus
		11.97	21	-7	28	no label
		11.88	21	-13	28	no label
		11.86	-60	-34	22	L Superior Temporal Gyrus
		11.37	-21	-31	64	L Postcentral Gyrus
		11.18	-6	-40	34	L Midcingulate Cortex
		11.09	-6	-46	34	L Midcingulate Cortex
		11.08	60	-1	7	R Rolandic Operculum
		10.85	39	5	13	R Insula Lobe
		10.50	-27	-25	67	L Precentral Gyrus
		10.43	-30	-28	64	L Precentral Gyrus
		10.43	30	-13	7	R Putamen
		10.42	63	-10	10	R Rolandic Operculum
		10.39	6	-49	22	R Precuneus
		10.34	57	-22	22	R Rolandic Operculum
		10.33	18	29	4	no label
	183	9.05	-51	32	10	L Inferior Frontal Gyrus p. Triangularis
	83	8.35	-45	-76	28	L Angular Gyrus
		6.42	-39	-76	37	L Middle Occipital Gyrus
	140	8.24	-27	-94	1	L Middle Occipital Gyrus
	227	7.98	-9	62	25	L Superior Medial Gyrus
		7.80	0	65	7	L Superior Medial Gyrus
		7.68	-3	56	4	L Superior Medial Gyrus
	55	7.29	57	32	7	R Inferior Frontal Gyrus p. Triangularis
	27	6.56	-27	-43	-8	L ParaHippocampal Gyrus
**TP2**						
	8281	12.94	30	-7	4	R Putamen
		12.06	-30	-13	4	L Putamen
		11.11	-60	-34	19	L Superior Temporal Gyrus
	241	7.14	21	-58	-23	R Cerebelum VI
		6.18	3	-64	-17	Cerebellar Vermis 6
		5.85	18	-46	-23	R Cerebelum IV-V
	38	6.64	-30	35	-11	L Inferior Frontal Gyrus p. Orbitalis
		5.93	-39	29	-14	L Inferior Frontal Gyrus p. Orbitalis
	45	6.55	-6	62	-5	L Mid Orbital Gyrus

		5.86	6 62 -5	R Mid Orbital Gyrus
	30	5.86	-54 32 13	L Inferior Frontal Gyrus p. Triangularis
	20	5.80	-18 32 13	no label
	10	5.53	-3 -46 -26	no label
**TP3**				
	9490	12.93	21 -4 28	no label
		12.73	-60 -31 22	L Superior Temporal Gyrus
		11.13	21 8 25	no label
	413	10.34	21 -52 -23	R Cerebelum VI
		8.20	6 -64 -14	Cerebellar Vermis 6
		7.40	-24 -58 -23	L Cerebelum VI
	213	7.26	-3 65 1	L Superior Medial Gyrus
		6.79	-6 62 16	L Superior Medial Gyrus
		6.20	-12 47 40	L Superior Frontal Gyrus
	42	7.18	30 -4 -23	R Hippocampus
	56	6.83	-51 32 4	L Inferior Frontal Gyrus p. Triangularis
**Negative > positive learning**				
**TP1**				
	3701	16.45	6 26 37	R Midcingulate Cortex
		15.40	30 23 1	no label
		15.31	6 20 46	R Posterior-Medial Frontal Gyrus
	761	14.29	54 -49 37	R Inferior Parietal Lobule
		11.62	54 -43 52	R Inferior Parietal Lobule
	108	11.56	-30 23 -2	L Insula Lobe
	104	7.61	-27 47 13	L Middle Frontal Gyrus
	196	7.53	-45 -43 43	L Inferior Parietal Lobule
		7.36	-51 -49 46	L Inferior Parietal Lobule
		7.04	-51 -55 40	L Inferior Parietal Lobule
	111	7.24	-9 -91 10	L Calcarine Gyrus
	36	7.01	6 -67 52	R Precuneus
	11	6.90	48 -25 -5	R Superior Temporal Gyrus
	77	6.60	-42 23 40	L Middle Frontal Gyrus
	17	6.23	-9 -64 49	L Precuneus
**TP2**				
	3213	16.16	6 26 40	R Midcingulate Cortex
		13.70	33 20 -5	no label
		11.04	42 2 31	R Precentral Gyrus
	190	12.28	-30 23 -5	L Insula Lobe
	421	10.08	-6 -94 16	L Cuneus
		9.99	-9 -91 4	L Calcarine Gyrus
	552	9.63	51 -46 46	R Inferior Parietal Lobule
		6.30	36 -58 49	R Angular Gyrus
	131	8.68	15 17 7	R Caudate Nucleus

		7.95	12 11 13	R Caudate Nucleus
		7.13	12 5 1	no label
	87	7.61	-9 8 10	L Caudate Nucleus
		6.82	-15 20 7	L Caudate Nucleus
		6.04	-9 5 1	no label
	52	6.71	-48 -43 46	L Inferior Parietal Lobule
	99	6.39	-36 -70 -17	L Fusiform Gyrus
**TP3**				
	2468	13.64	30 23 -2	no label
		12.34	6 23 43	R Superior Medial Gyrus
		12.22	6 17 52	R Posterior-Medial Frontal Gyrus
	147	11.08	-30 23 -2	L Insula Lobe
		6.89	-42 17 7	L Inferior Frontal Gyrus p. Triangularis
	122	8.93	-9 -91 10	L Calcarine Gyrus
	216	8.75	54 -49 37	R Inferior Parietal Lobule
		8.74	51 -43 49	R Inferior Parietal Lobule
		6.32	39 -58 49	R Angular Gyrus
	407	8.61	-42 -64 -5	L Inferior Occipital Gyrus
		8.14	-42 -85 10	L Middle Occipital Gyrus
		7.84	-36 -58 -11	L Fusiform Gyrus
	210	8.53	15 14 10	R Caudate Nucleus
		7.89	9 -7 4	R Thalamus
		6.48	0 -28 -2	no label
	161	7.98	42 -61 -11	R Inferior Temporal Gyrus
		6.60	36 -43 -20	R Fusiform Gyrus
	69	6.80	-12 14 13	L Caudate Nucleus
		5.88	-9 5 -2	L Pallidum
	29	6.54	-48 -43 49	L Inferior Parietal Lobule
	16	6.38	3 -25 -23	no label
	22	6.24	-45 2 31	L Precentral Gyrus

Note: clusters > 10 voxels are reported. We reported the first 3 local maxima. For clusters > 10000 voxels, we reported the first 10 local maxima

Supplementary Table 2: N, Mean and SD values for ROI activity for sensitivity to informative value (learning > application) and sensitivity to valence (positive > negative learning) at each time point.

	TP1			TP2			TP3		
**Sensitivity to informative value**	**N**	**Mean**	**SD**	**N**	**Mean**	**SD**	**N**	**Mean**	**SD**
Dorsal caudate	271	1.50	1.19	233	1.77	1.42	232	0.98	0.73
Ventral caudate	271	0.98	1.29	233	1.52	1.41	232	0.86	0.73
Nucleus accumbens	271	-0.31	1.09	233	0.04	1.18	232	-0.26	0.58
**Sensitivity to valence**									
Dorsal caudate	271	-0.75	1.72	233	-0.58	1.74	232	-0.32	1.06
Ventral caudate	271	-0.51	1.82	233	-0.63	1.90	232	-0.34	1.07
Nucleus accumbens	271	0.51	1.57	233	0.28	1.52	232	1.71	0.76

Supplementary Table 3: Intra-Class-Correlation values for neural sensitivity to informative value (learning > application) and sensitivity to valence (positive > negative learning). We used a two-way mixed model with absolute agreement and we reported the average measure.

**Brain region**	**Informative Value**	**Valence**
Dorsal caudate	.402	.100
Ventral caudate	.414	.248
Nucleus accumbens	.438	.231

Supplementary Table 4: Predicting learning performance above a quadratic effect of age from neural sensitivity to valence (positive > negative learning) (*N*=731). More activity in ventral caudate for negative compared to positive learning predicted learning performance. When adding striatal activity to the model we used the regression residuals from the best age model for that region.

**Model parameter**	***B***	**SE**	***t***	***p***
Intercept	94.62	0.21	457.42	<.001
Age^1	48.13	5.13	9.38	<.001
Age^2	-30.08	4.75	-6.33	<.001
**Ventral caudate**	-0.34	0.16	-2.11	.036
